# Increased Level of Vascular Endothelial Growth Factors by 4-hexylresorcinol is Mediated by Transforming Growth Factor-β1 and Accelerates Capillary Regeneration in the Burns in Diabetic Animals

**DOI:** 10.3390/ijms21103473

**Published:** 2020-05-14

**Authors:** Dae-Won Kim, You-Young Jo, Umberto Garagiola, Je-Yong Choi, Yei-Jin Kang, Ji-Hyeon Oh, Seong-Gon Kim

**Affiliations:** 1 Department of Oral Biochemistry, College of Dentistry, Gangneung-Wonju National University, Gangneung 28644, Korea; kimdw@gwnu.ac.kr; 2 Sericultural and Apicultural Division, National Institute of Agricultural Science, Rural Development Administration, Wanju 55365, Korea; yyjo@korea.kr; 3 Biomedical, Surgical and Oral Sciences Department, Maxillofacial and Dental Unit, School of Dentistry, University of Milan, 20122 Milan, Italy; umberto.garagiola@unimi.it; 4 School of Biochemistry and Cell Biology, BK21 Plus KNU Biomedical Convergence Program, Skeletal Diseases Analysis Center, Korea Mouse Phenotyping Center (KMPC), Kyungpook National University, Daegu 41944, Korea; jechoi@knu.ac.kr; 5 Department of Oral and Maxillofacial Surgery, College of Dentistry, Gangneung-Wonju National University, Gangneung 28644, Korea; kyj292@hanmail.net (Y.-J.K.); haruna348@naver.com (J.-H.O.)

**Keywords:** 4-hexylresorcinol, HUVEC, diabetes mellitus, TGF-β1, angiogenesis

## Abstract

4-Hexyl resorcinol (4HR) is an organic compound and has been used in skin care application. 4HR is an M2-type macrophage activator and elevates vascular endothelial growth factor (VEGF) expression via the hypoxia-inducible factor (HIF)-independent pathway. As endothelial cells are important in wound healing, the human umbilical vein endothelial cells (HUVECs) were treated with 4HR, and changes in VEGF-A, -C, and transforming growth factor-β1 (TGF-β1) expression were investigated. The administration of 4HR increased the expression level of VEGF-A, -C, and TGF-β1. The application of TGF-β1 protein also increased the expression level of VEGF-A and -C. Knockdown with small interfering RNA (siRNA) targeting to TGF-β1 and the selective chemical inhibition (A83-01) to ALK5 confirmed the involvement of the TGF-β signaling pathway in the 4-HR-mediated VEGFs expression. 4HR application in a burn model of diabetic rats demonstrated an increased level of angiogenic proteins with wound healing. Compared to sericin application, the 4HR application group showed more prominent capillary regeneration. Collectively, 4HR activated TGF-β1/ALK5/VEGFs signaling in endothelial cells and induced vascular regeneration and remodeling for wound healing.

## 1. Introduction

Angiogenesis is important for uneventful wound healing. Impaired angiogenesis is the main cause of delayed wound healing. Many types of wound-healing disorders are associated with impaired angiogenesis, such as drug-induced osteonecrosis [1], radiation-induced jaw bone necrosis [2], and skin ulcers in patients with diabetes mellitus (DM). DM hinders skin wound healing because of a poor vascular network [3]. Chronic skin ulcers are frequently found in the foot of DM patients, and their incidence is approximately 25% of DM patients [4]. Among them, many patients suffered from foot amputation or recurrent ulcers [5]. In the case of burns, the overall complication rate is approximately 90% of DM patients [6]. The time to complete epithelialization is much longer in DM patients [6]. Accordingly, DM burn patients stay in the hospital for much longer periods [7].

Several types of cells are involved in angiogenesis. Macrophages are important cells for angiogenesis and wound healing [8]. Macrophages orchestrate the healing process by secreting cytokines. Cytokines secreted by macrophages are responsible for the inflammatory phase to remodeling phase in wound healing [8]. The type of macrophage is grossly classified as M1 and M2. M1-type macrophages are important in the inflammatory phase [9]. M1-type macrophages secrete vascular endothelial growth factors (VEGFs) and dilate vessels with increased permeability [9,10]. Sericin is a protein originated from the silkworm cocoon and the degumming product of the silk industry [11]. Sericin increases M1 markers including tumor necrosis factor-α (TNF-α) and hypoxia-inducible factor-1α (HIF-1α) [11]. TNF-α helps VEGF via HIF-1α to modulate vascular events in the inflammatory phase [10]. Therefore, sericin application may help wound healing via vasodilatation with increased permeability. However, prolonged vessel dilatation with increased permeability may result in chronic inflammation and will be healed as highly fibrotic tissue. Therefore, wound remodeling and capillary regeneration should be followed by an inflammatory phase. M2-type macrophages are important in the remodeling phase [9,12]. M2-type macrophages secrete transforming growth factor-β1 (TGF-β1) [9]. TGF-β1 helps VEGF to increase capillary regeneration [13]. Our recent study demonstrated that 4-hexyl resorcinol (4HR) increases VEGF-A and -C expression in RAW264.7 cells [11]. However, the relationship between the elevated expression level of VEGF and TGF-β1 has not been clarified.

Endothelial cells are also important cells for angiogenesis. In the diabetic endothelial damage, the production of reactive oxygen species (ROS) is increased [14] and the signaling pathway of insulin is altered [15]. Many genes associated with inflammation and vascular permeability are upregulated in the endothelial cells of streptozotocin (STZ)-injected mice [16]. 4HR inhibits ROS production and has anti-inflammatory effects [11]. Therefore, 4HR administration in a DM animal model would be helpful for demonstrating its effect on wound healing and capillary regeneration. As sericin has been used for the treatment of burns [17] and is an M1 inducer [11], a sericin application group could be a positive control for the 4HR application group in a DM burn model.

Our hypothesis in this study was that TGF-β1 played a key role in 4HR-induced activation of the angiogenesis-associated signaling pathway in human umbilical vein endothelial cells (HUVECs). The administration of 4HR increases TGF-β1 expression in RAW264.7 cells [18] and Saos-2 cells [19]. However, previous studies have not demonstrated the relationship between TGF-β1 expression and activation of angiogenesis. To confirm this hypothesis, Western blotting with an inhibition assay was done with TGF-β1 and its signal blocker. In addition, the capillary regeneration pattern was examined in burn wounds of the DM model. For the comparison of a wound healing pattern, sericin, an M1 activator, was used in the animal model.

## 2. Results

### 2.1. HR-Induced VEGF Expression in HUVECs Was Reduced by TGF-β1 siRNA

**Figure 1:**
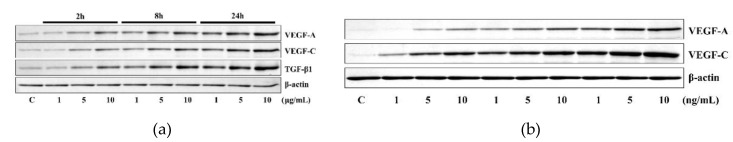
Western blot analysis. (**a**) The application of 4HR increased the expression of VEGF-A, VEGF-C, and TGF-β1. (**b**) The application of TGF-β1 also increased the expression of VEGF-A and VEGF-C. Full-length gels and blots are included in Figures S1 and S2.

The application of 1 to 10 μg/mL 4HR increased the expression of VEGF-A, VEGF-C, and TGF-β1 in HUVECs. These results indicated that 4HR induced TGF-β1, VEGF-A, and VEGF-C simultaneously (Figure 1a). The application of 1 to 10 ng/mL of TGF-β1 increased the expression of VEGF-A and VEGF-C in HUVECs depending on time (2, 8, and 24 h) and dose (1, 5, and 10 ng). These results indicated that TGF-β1 efficiently induced the expressions of VEGF-A and VEGF-C even in minute doses (1, 5, 10 ng) (Figure 1b).

**Figure 2:**
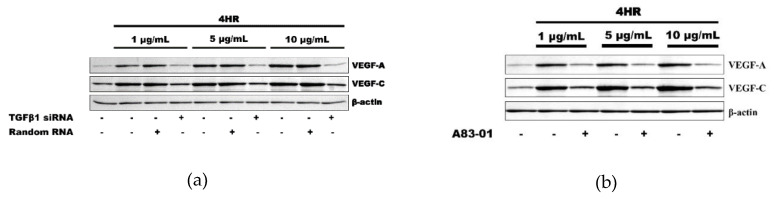
Inhibition assay. (**a**) The application of TGF-β1 small interfering RNA (siRNA) inhibited the expression of VEGF-A and VEGF-C, which was induced by 4HR application. (**b**) ALK5 inhibitor, A83-01, also showed a similar effect to that of TGF-β1 siRNA on the VEGFs expression by 4HR in human umbilical vein endothelial cells (HUVECs). Full-length gels and blots are included in Figures S3 and S4.

As a loss-of-functional experiment, 4HR-induced VEGFs expressions were decreased by the application of TGF-β1 siRNA, indicating involvement of TGF-β1 expression in 4HR-induced VEGF-A and VEGF–C expressions (Figure 2a). Moreover, A83-01, an ALK5 inhibitor, also decreased the 4HR-mediated expression of VEGF-A and VEGF–C. These results indicated that 4HR-induced VEGF-A and VEGF–C expressions can be regulated by modulation of the TGFβ/ALK5 signaling pathway (Figure 2b).

### 2.2. HR-Induced Capillary Regeneration in a Diabetic Rat Model

**Figure 3:**
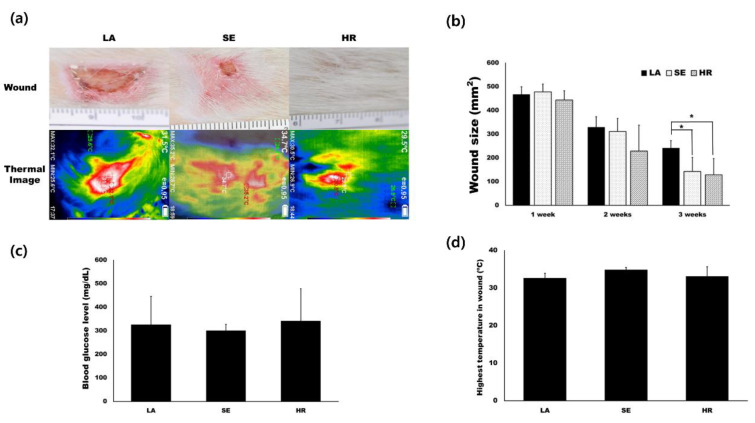
Animal experiments. (**a**) Images for residual wound and thermogram at 3 weeks after injury. The images at different time points are available in Figure S5. (**b**) Residual wound size was significantly different at 3 weeks after injury. Compared to the ointment base only group, the 4HR and sericin groups showed a significantly small size of the residual wound (*p* = 0.022 and 0.049, respectively). (**c**) The average blood glucose level was approximately 300 mg/dL in all groups and there was no significant difference among groups (*p* > 0.05). (**d**) In the case of surface temperature, the sericin group was the highest and the 4HR group was followed (LA; lanolin only, SE; sericin, HR; 4HR).

Based on in vitro results, we tested 4HR’s effect on capillary regeneration using an STZ-induced type I diabetic wound healing rat model. We prepared ointment base only (LA), sericin (SE), and 4HR (HR) groups as indicated in the Materials and Methods. Residual wound size was significantly different at 3 weeks after injury (Figure 3a,b, *p* = 0.015). Compared to the ointment base only group, the 4HR and sericin groups showed a significantly small size of residual wound (*p* = 0.022 and 0.049, respectively). The average blood glucose level was approximately 300 mg/dL in all groups, and there was no significant difference among groups (Figure 3c, *p* > 0.05). In the case of surface temperature, the sericin group was the highest, followed by the 4HR group (Figure 3d).

**Figure 4:**
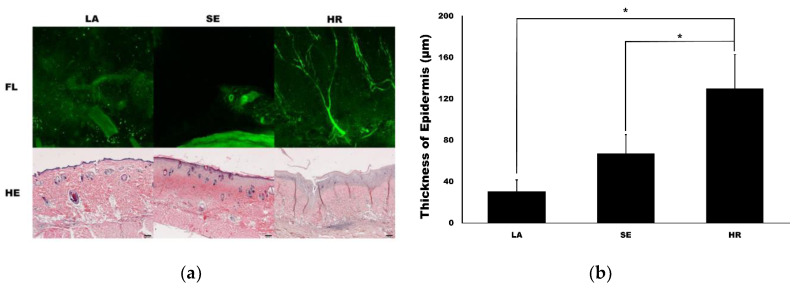
Capillary and epidermis regeneration. (**a**) The images from confocal microscopy (FL) and light microscopy (hematoxylin and eosin (HE)). Movie clips for confocal images are available in supplementary data. The regeneration of the capillary was prominent in the 4HR-administered group (HR) compared to the ointment base only group (LA) and sericin-administered group (SE) in confocal images (original magnification ×100). Epithelial regeneration shown in light microscopic views was also different among groups (original magnification ×40, hematoxylin and eosin stain). (**b**) The thickness of the epidermis was significantly different among groups (*p* < 0.001). The thickness of the epidermis was significantly higher in the HR group than those in the LA and SE groups (* *p* < 0.05).

Confocal microscopy imaging demonstrated that capillary regeneration was prominent in the 4HR-administered group compared to the other groups (Figure 4a). Interestingly, vessel dilatation was prominent in the sericin-administered group. In HE staining, the thickness of the 4HR group was larger than the other groups. When measuring the thickness of the epidermis, the LA group was 30.72 ± 11.04 µm. It was 67.10 ± 18.39 µm and 130.13 ± 32.65 µm in the SE group and HR group, respectively (Figure 4b). The difference among groups was statistically significant (*p* < 0.001). In post-hoc testing, the HR group was significantly higher than those in the SE and LA groups (*p* = 0.003 and <0.001, respectively).

**Figure 5:**
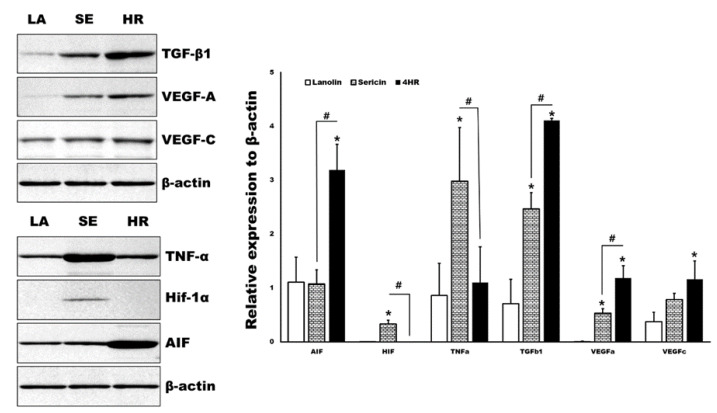
Western blot analysis for tissue samples. TGF-β1 and apoptosis-inducing factor (AIF) were significantly highly expressed in the 4HR-administered group (HR) compared to the ointment base only group (LA) and sericin-administered group (SE) (*p* < 0.05). TNF-α and Hif-1α were significantly highly expressed in the SE group compared to the LA and HR groups (*p* < 0.05). Full-length gels and blots are included in Figure S6. (TGF-β1: Transforming growth factor-β1, VEGF: Vascular endothelial growth factor, TNF-α: Tumor necrosis factor-α, Hif: Hypoxia-inducible factor, AIF: Apoptosis inducible factor. * *p* < 0.05 compared to LA group. # *p* < 0.05 between SE and HR group).

By Western blot analysis, various angiogenic makers and upstream regulators of tissue samples were measured in parallel (Figure 5). The expression levels of TGF-β1 and apoptosis-inducible factor (AIF) were significantly higher in the 4HR administered groups compared to the other groups (*p* < 0.05). Both the 4HR and sericin groups showed higher expression levels of VEGF-A compared to the ointment base only group (*p* < 0.05). In the sericin-administered group, the expression levels of TNF-α and Hif-1α were significantly higher than those of the other groups (*p* < 0.05).

**Figure 6:**
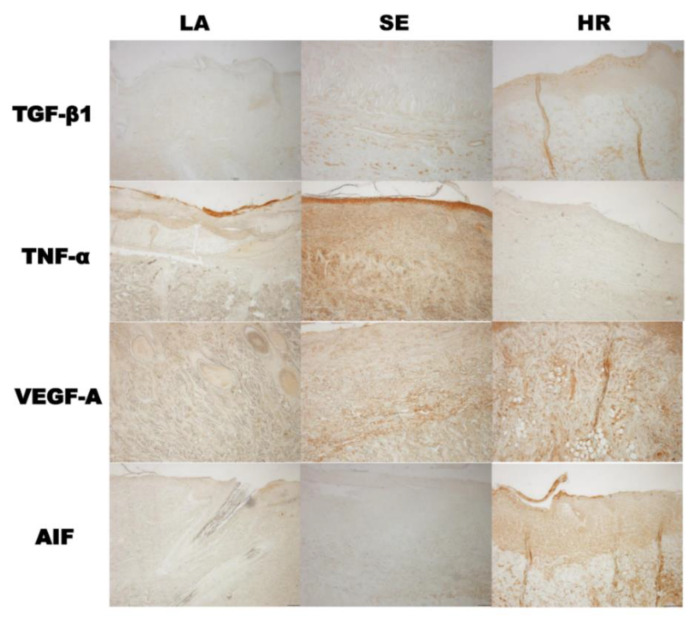
Immunohistochemical findings. The results of immunohistochemistry were also in accordance with those of tissue Western blot. TGF-β1 and AIF were highly expressed in the 4HR-administered group and TNF-α in the sericin group (LA: Lanolin ointment base only, SE: Sericin-administered group, HR: 4HR-administered group, original magnification ×100 without counterstaining).

The results of immunohistochemistry were also in accordance with those of tissue Western blot. TGF-β1 and AIF were highly expressed in 4HR administered groups and TNF-α in the sericin group (Figure 6).

## 3. Discussion

In the previous study, we found that 4HR induced potent de novo angiogenesis in both in vitro and in vivo experiments [11]. 4HR treatment increased VEGF expression in RAW264.7 cells, and it is HIF-independent [11]. The present study explored the molecular mechanism of 4HR-induced angiogenesis in HUVECs and observed that 4HR-treated HUVECs showed dominant expressions of TGF-βs concurrently with upregulation of VEGFs (Figure 1 and Figure 2). The inhibition of ALK5 has been known to block the Smad pathway [20]. In this study, we showed that A83-01 as an ALK5 inhibitor decreased the 4HR-mediated expression of VEGF-A and VEGF-C in HUVECs. Therefore, it is suggested that 4HR-induced angiogenesis in HUVECs is characteristic with serial activations of cellular angiogenetic factors in the TGF-βs/SMADs/VEGFs pathways independent from the ordinary angiogenesis transcription factor (HIF-1α). 4HR-induced VEGF-A and -C expressions were reduced by TGF-β1 siRNA or A83-01 treatment (SMAD inhibitor) in a time- and dose-dependent manner (Figure 2). Compared to M1 activator (sericin), 4HR showed a significantly higher epithelial thickness with prominent capillary regeneration in the STZ-induced DM animal model (*p* < 0.05; Figure 4). The expressions of HIF-1α and TNF-α were significantly higher in the sericin group compared to the 4HR group (*p* < 0.05; Figure 5). However, the 4HR group showed elevated expression of TGF-β1 and AIF (*p* < 0.05; Figure 5).

As the 4HR has a long alkyl group, it is strongly hydrophobic and able to bind some proteins. As a result, 4HR adhered proteins change their conformation into the inactive status [21]. According to our 4HR adhering assay [18], TNF-α, PDGF-A, and pAkt1/2/3 are strongly adherent to 4HR. However, TGF-β1, apoptosis-related proteins, and angiogenesis-related proteins (angiogenin, VEGF-A, VEGF-C) are barely adherent (<5%) to 4HR-coated beads [18]. In this study, TNF-α expression was reduced by 4HR treatment, but TGF-β1, VEGF-A, and AIF expression was increased (Figure 1, Figure 5 and Figure 6, respectively). This 4HR-induced cellular apoptosis slowly progressed with no activation of nuclear factor (NF)-kB signaling and compensated by stimulating TGF-β1 production in HUVECs (Figure 1). To confirm the signaling pathway of TGF-βs/SMADs/VEGFs, protein inhibitory assays were performed using siRNA-targeting TGF-β1 and selective inhibitor A83-01 of ALK5,18, which is a receptor for TGF-β1 and an upstream protein of the SMAD pathway (Figure 2). 4HR coincidently increased the expression level of TGF-β1 and VEGFs in Western blot, and 4HR-induced VEGF-A and -C expressions were reduced by TGF-β1 siRNA or A83-01 treatment in a time- and dose-dependent manner (Figure 1 and Figure 2). Therefore, it is assumed that 4HR activates TGF-βs/SMADs/VEGFs signaling and induces vascular regeneration and remodeling for wound healing.

In particular, the overexpression of TGF-β1 in 4HR-treated HUVECs might be ascribed to the increase in apoptosis via Fas-mediated signaling, and the dominant TGF-β1 expression might induce the protein expressions of M2 macrophage polarization proteins for the cross-talk to macrophages, which may subsequently stimulate wound-healing procedures. TNF-α, interleukin (IL)-6, leukotriene A4 hydrolase, and C-X-C chemokine receptor type 4 are M1 macrophage polarization proteins [9,22]. However, IL-10, lysozyme, granzyme B, and macrophage colony-stimulating factor cytokines are M2 macrophage polarization proteins [9,22]. M1 macrophage polarization proteins reduced their expression, but M2 macrophage polarization proteins were increased by 4HR treatment in HUVECs according to our immunoprecipitation assay [23]. Among 4HR-induced angiogenic effects, M2 macrophage polarization proteins will be more greatly amplified in in vivo animal experiments, where macrophages can be infiltrated, than in in vitro cell culture. 4HR-treated animals expressed a higher level of TGF-β1 and AIF than lanolin and sericin-treated animals (Figure 5 and Figure 6). The upregulation of M2 macrophage polarization cytokines might autonomously stimulate HUVECs to undergo cytological changes appropriate for angiogenesis, subsequently followed by HUVEC differentiation via TGF-βs/SMADs/VEGFs signaling in vitro. Our previous study reported that 4HR induced a strong wound healing effect with TNF-α suppression [24]. 4HR also induced de novo angiogenesis associated with M2 macrophage infiltration in in vivo animal experiments [11]. In contrast to 4HR treatment, sericin-M1 activator-treated animals showed upregulation of TNF-α (M1 macrophage polarization protein) (Figure 5 and Figure 6). Accordingly, sericin-treated animals showed vessel dilatation, such as inflammatory phase and 4HR-treated animal capillary regeneration. Inflammation increases body temperature via vessel dilatation [25]. Thermography can detect the inflammatory features via checking surface temperature [25]. Surface temperature was highest in the sericin-treated group, and the 4HR-treated group was the second via capillary regeneration (Figure 3 and Figure 4).

In this animal study, capillary regeneration and epithelial thickness were higher in the 4HR-treated group (Figure 4). Epithelial thickness and capillary count are positively correlated [26]. 4HR increases epithelial differentiation markers, such as keratin 10 and involucrin [27]. Normal epithelial cells are important in the formation of tissue-engineered capillaries [28]. The inhibitor of ROS production increases epithelial proliferation with capillary regeneration [29]. 4HR inhibits ROS production [11]. TGF-β1 is mainly expressed in regenerated endothelial cells, epithelial cells, and fibroblasts [30]. According to a previous study [31], 4HR was suspected to be xenoestrogen. Although estrogen can increase angiogenesis [32], the effect and signaling pathway of 4HR are different from those of estrogen [33]. Accordingly, TGF-β1 is a key cytokine in skin wound healing, and 4HR increased its level of expression in this study.

## 4. Materials and Methods

### 4.1. HUVEC Culture

HUVECs (Lonza, Walkersville, MD, USA) were purchased and cultured. The medium was an endothelial cell basal medium containing supplements (EGMTM-2, Clonetics^®^, Lonza, Walkersville, MD, USA). Cells were cultured in 5% CO_2_ at 37.5 °C. Mycoplasma tests were done on a regular basis to ensure that only mycoplasma-free cells were assayed.

### 4.2. Western Blot and Inhibitory Assay

When HUVECs were grown, approximately 70% confluent cells were treated with 1, 5, and 10 µg/mL 4HR for 2, 8, or 24 h; control cells were treated with 1 mL of normal saline. Cultured cells were harvested with protein lysis buffer (PRO-PREP^TM^, iNtRON Biotechnology INC, Sungnam, Korea) and underwent Western blotting. The quantification of proteins was done as described previously [11]. To assess the effect of TGF-β1 application on the expression of VEGF-A and VEGF-C, 1–10 ng/mL of rhTGF-β1 protein was applied and proteins were collected after 2, 8, and 24 h. TGF-β1 siRNA was purchased from Bioneer (Chungwon, Korea). The optimal inhibitory concentration of siRNA was tested before final application and set as 20 pg/mL (Figure S7). After pretreatment of TGF-β1 siRNA, 4HR was applied with 1 to 10 μg/mL, and the protein was collected 24 h after 4HR application. The same concentration of random RNA application was set as a positive control. The level of VEGF-A and VEGF-C was checked. A83-01 is an inhibitor of ALK5. The optimal concentration of A83-01 was determined in the recommended range of optimal concentration suggested by the manufacturer and confirmed by the inhibitory experiment. HUVEC cells were pretreated with dimethyl sulfoxide (DMSO) or DMSO + 12 nM A83-01. Then, the cells were treated with 1–10 μg/mL of 4HR, cultured for 24 h, and subjected to Western blot analysis for VEGF-A and VEGF-C.

### 4.3. Animal Experiments

The animal experiments were approved by the Institutional Animal Care and Use Committee, Gangneung-Wonju National University (GWNU-2019-12, approval date 22 May 2019). All methods were carried out in accordance with relevant guidelines and regulations. Twenty male Wister rats (10 weeks old with 250–300 g body weight) were purchased from Samtako (Seoul, Korea). After a 1 week adaptation period, the drug was injected for inducing type I DM. For the preparation of solvent for STZ (Sigma-Aldrich, St. Louis, MO, USA), 1.47 g sodium citrate was solubilized in 50 mL of distilled water (DW), and the pH was set as 4.5. The dosage of STZ was determined by the body weight, and 50 mg/kg was administered into the tail vein under inhalation anesthesia. Blood glucose level was checked 3 days after STZ injection under 8 h fasting conditions. The animals that showed at least 150 mg/dL of blood glucose level were selected for further study. A deep burn animal model was referenced from our previous publication [24]. Accordingly, 18 animals were included. The group in which ointment alone was applied was used as the negative control. Sericin is an M1 inducer [11] and assumed to be different in capillary regeneration than the M2 inducer applied group. Accordingly, the group receiving ointment application containing 20 wt.% hydrated sericin was used as a positive control. The experimental group was treated with 2 wt.% 4HR ointment. A brief summary of the animal study is shown in Figure S8. All procedures were done under medication-induced general anesthesia. After removing hair on the back, the target area was painted with 10% povidone-iodine. The design of the third-degree deep burn wound was referenced from our previous publication [24]. Briefly, the hotplate (size: 2 cm × 2 cm, temperature: 170 °C) was placed on the de-haired back skin for 2 s. One ointment among 3 different kinds of ointment was applied for each group. To control postoperative pain and infection, 1 mg/kg of gentamicin (Kukje Pharm, Seongnam, Korea) and 0.5 mL/kg of pyrin (Green Cross Veterinary Products, Yongin, Korea) were injected. Ointment was applied every 3 days. Each animal received only one burn wound.

The wound size was evaluated by digital photographs taken at 1, 2, and 3 weeks. The wound size was measured with Sigma Pro^®^ (SPSS Inc., Chicago, IL, USA). Thermography was taken at 3 weeks. Before killing animals, 100 μg of DyLight^®^ 488 was injected into the heart for visualization of vessels. Skin specimens were divided into 3 pieces. One group of samples were sent for histological analysis. Another group of samples were stored in a deep freezer (−80 °C) and used for Western blot analysis. The other samples underwent a tissue transparent procedure for the analysis of confocal microscopy.

### 4.4. Tissue Transparent Procedure and Confocal Microscopic Examination

The tissue transparent procedure was done with a kit (Binaree, Daegu, Korea). Briefly, skin samples of approximately 5 mm thickness were fixed in paraformaldehyde for 8 h at 4 °C. Then, the samples were placed in the tissue clearing reagent for a week. After washing, the samples were examined with confocal microscopy (FV3000, Olympus, Tokyo, Japan). The thickness of each slice was set as 3 μm at an original magnification of ×100. Thirty sliced images along the z-axis were reconstructed as a 3-dimensional movie.

### 4.5. Histological Analysis and Western Blot for Tissue Samples

Back skin samples for histological analysis were fixed and treated in the tissue processor for the preparation of paraffin blocks. The paraffin block sections were used for hematoxylin and eosin (HE) stain. Immunohistochemical staining was done for TNF-α, TGF-β1, VEGF-A, and apoptosis-inducing factor (AIF). The purchased antibodies were anti-TNF-α antibody (Abcam, Cambridge, UK), anti-TGF-β1 antibody (Santa Cruz Biotechnology, Santa Cruz, CA, USA), anti-VEGF-A antibody (Santa Cruz Biotechnology), and anti-AIF antibody (Santa Cruz Biotechnology) at a dilution of 1:50. The detailed procedure was in accordance with our previous publication [11]. Sections stained without primary antibody were used as negative controls.

Three skin samples in the deep freezer were used for Western blot. Total proteins were extracted from the skin. After measuring protein concentration, Western blot for TGF-β1, VEGF-A, VEGF-C, TNF-α, hypoxia inducible factor-1α (HIF-1α), AIF, and β-actin were done. The manufacturers for primary antibodies were the same as those used in immunohistochemistry. Anti-HIF-1α antibody was purchased from Santa Cruz Biotechnology. The subsequent procedure for Western blot was in accordance with that in the cellular experiment. The expression level of each protein in the gel photo was analyzed using Sigma Pro^®^ (SPSS Inc., Chicago, IL, USA). The relative expression level of each protein was calculated by comparing β-actin expression level.

### 4.6. Statistical Analysis

The difference in the protein expression of tissue samples was evaluated by analysis of variance. The Bonferroni method was used as the post-hoc test. The level of significance was set as *p* < 0.05.

## 5. Conclusions

Collectively, 4HR-induced angiogenic factors (VEGFs) were controlled by TGF-β1 expression and subsequent activation of SMADs/VEGFs signaling. Therefore, it is assumed that 4HR activates TGF-βs/SMADs/VEGFs signaling and induces vascular regeneration and remodeling for wound healing. In particular, 4HR (M2 activator)-treated DM animals showed higher epithelial thickness with prominent capillary regeneration compared to the sericin (M1 activator)-treated group. The dominant TGF-β1 expression might induce the protein expressions of M2 macrophage polarization proteins in endothelial cells, which subsequently stimulate wound-healing procedures via cooperation with macrophages.

## Supplementary Materials

The following are available online at https://www.mdpi.com/1422-0067/21/10/3473/s1. Figure S1. Full-length blot of Figure 1A. Figure S2. Full-length blot of Figure 1B. Figure S3. Full-length blot of Figure 2A. Figure S4. Full-length blot of Figure 2B. Figure S5. The images for burn wound at different time point. Figure S6. Full-length blot of Figure 5. Figure S7. Determination of optimal concentration of TGF-β1 siRNA. Figure S8. CORT chart for the animal experiment.

## References

[1] Son H.J., Kim J.W., Kim S.J. (2019). Pharmacoepidemiology and clinical characteristics of medication-related osteonecrosis of the jaw. Maxillofac. Plast. Reconstr. Surg..

[2] Jung H.M., Lee J.E., Lee S.J., Lee J.T., Kwon T.Y., Kwon T.G. (2018). Development of an experimental model for radiation-induced inhibition of cranial bone regeneration. Maxillofac. Plast. Reconstr. Surg..

[3] Okonkwo U.A., DiPietro L.A. (2017). Diabetes and wound angiogenesis. Int. J. Mol. Sci..

[4] Alavi A., Sibbald R.G., Mayer D., Goodman L., Botros M., Armstrong D.G., Woo K., Boeni T., Ayello E.A., Kirsner R.S. (2014). Diabetic foot ulcers: Part, I. Pathophysiology and prevention. J. Am. Acad. Dermatol..

[5] Jeffcoate W.J., Harding K.G. (2003). Diabetic foot ulcers. Lancet.

[6] Goutos I., Nicholas R.S., Pandya A.A., Ghosh S.J. (2015). Diabetes mellitus and burns. Part II-outcomes from burn injuries and future directions. Int. J. Burn Trauma.

[7] Duke J.M., Randall S.M., Fear M.W., Boyd J.H., O’Halloran E., Rea S., Wood F.M. (2016). Increased admissions for diabetes mellitus after burn. Burns.

[8] Kim S.G. (2020). Immunomodulation for maxillofacial reconstructive surgery. Maxillofac. Plast. Reconstr. Surg..

[9] Chen Z., Klein T., Murray R.Z., Crawford R., Chang J., Wu C., Xiao Y. (2016). Osteoimmunomodulation for the development of advanced bone biomaterials. Mater. Today.

[10] Kim K.W., Lee S.J., Kim J.C. (2017). TNF-α upregulates HIF-1α expression in pterygium fibroblasts and enhances their susceptibility to VEGF independent of hypoxia. Exp. Eye Res..

[11] Jo Y.Y., Kim D.W., Choi J.Y., Kim S.G. (2019). 4-Hexylresorcinol and silk sericin increase the expression of vascular endothelial growth factor via different pathways. Sci. Rep..

[12] Spiller K.L., Nassiri S., Witherel C.E., Anfang R.R., Ng J., Nakazawa K.R., Yu T., Vunjak-Novakovic G. (2015). Sequential delivery of immunomodulatory cytokines to facilitate the M1-to-M2 transition of macrophages and enhance vascularization of bone scaffolds. Biomaterials.

[13] Kariya T., Nishimura H., Mizuno M., Suzuki Y., Matsukawa Y., Sakata F., Maruyama S., Takei Y., Ito Y. (2018). TGF-β1-VEGF-A pathway induces neoangiogenesis with peritoneal fibrosis in patients undergoing peritoneal dialysis. Am. J. Physiol. Renal Physiol..

[14] Zou M.H., Cohen R., Ullrich V. (2004). Peroxynitrite and vascular endothelial dysfunction in diabetes mellitus. Endothelium.

[15] Ritchie S.A., Ewart M.A., Perry C.G., Connell J.M., Salt I.P. (2004). The role of insulin and the adipocytokines in regulation of vascular endothelial function. Clin. Sci..

[16] Maresh J.G., Shohet R.V. (2008). In vivo endothelial gene regulation in diabetes. Cardiovasc. Diabetol..

[17] Joob B., Wiwanitkit V. (2013). Silk sericin and burn wound. Arch. Dermatol. Res..

[18] Kim M.K., Yoon C.S., Kim S.G., Park Y.W., Lee S.K. (2019). Effects of 4-hexylresorcinol on protein expressions in RAW 264.7 cells as determined by immunoprecipitation high performance liquid chromatography. Sci. Rep..

[19] Choi K.H., Kim D.W., Lee S.K., Kim S.G., Kim T.W. (2020). The administration of 4-hexylresorcinol accelerates orthodontic tooth movement and increases the expression level of bone turnover markers in ovariectomized rats. Int. J. Mol. Sci..

[20] Tojo M., Hamashima Y., Hanyu A., Kajimoto T., Saitoh M., Miyazono K., Node M., Imamura T. (2005). The ALK-5 inhibitor A-83-01 inhibits Smad signaling and epithelial-to-mesenchymal transition by transforming growth factor-beta. Cancer Sci..

[21] Feng S., Song X.H., Zeng C.M. (2012). Inhibition of amyloid fibrillation of lysozyme by phenolic compounds involves quinoprotein formation. FEBS Lett..

[22] Shapouri-Moghaddam A., Mohammadian S., Vazini H., Taghadosi M., Esmaeili S.A., Mardani F., Seifi B., Mohammadi A., Afshari J.T., Sahebkar A. (2018). Macrophage plasticity, polarization, and function in health and disease. J. Cell Physiol..

[23] Kim M.K., Kim S.G., Lee S.K. (2020). 4-Hexylresorcinol-induced angiogenesis potential in human endothelial cells. Maxillofac. Plast. Reconstr. Surg..

[24] Ahn J., Kim S.G., Kim M.K., Kim D.W., Lee J.H., Seok H., Choi J.Y. (2016). Topical delivery of 4-hexylresorcinol promotes wound healing via tumor necrosis factor-α suppression. Burns.

[25] Ranosz-Janicka I., Lis-Święty A., Skrzypek-Salamon A., Brzezińska-Wcisło L. (2019). Detecting and quantifying activity/inflammation in localized scleroderma with thermal imaging. Skin Res. Technol..

[26] Eyuboglu A.A., Uysal C.A., Ozgun G., Coskun E., Markal Ertas N., Haberal M. (2018). The effect of adipose derived stromal vascular fraction on stasis zone in an experimental burn model. Burns.

[27] Kim S.G., Kim A.S., Jeong J.H., Choi J.Y., Kweon H. (2012). 4-hexylresorcinol stimulates the differentiation of SCC-9 cells through the suppression of E2F2, E2F3 and Sp3 expression and the promotion of Sp1 expression. Oncol. Rep..

[28] Rochon M.H., Fradette J., Fortin V., Tomasetig F., Roberge C.J., Baker K., Berthod F., Auger F.A., Germain L. (2010). Normal human epithelial cells regulate the size and morphology of tissue-engineered capillaries. Tissue Eng. Part A.

[29] Akçay M.N., Ozcan O., Gündoğdu C., Akçay G., Balik A., Köse K., Oren D. (2000). Effect of nitric oxide synthase inhibitor on experimentally induced burn wounds. J. Trauma.

[30] Wang X., Niu X., Cheng D. (1997). TGF-beta 1 gene expression in the healing process of skin wound in rat. Zhongguo Xiu Fu Chong Jian Wai Ke Za Zhi.

[31] Amadasi A., Mozzarelli A., Meda C., Maggi A., Cozzini P. (2009). Identification of xenoestrogens in food additives by an integrated in silico and in vitro approach. Chem. Res. Toxicol..

[32] Trenti A., Tedesco S., Boscaro C., Trevisi L., Bolego C., Cignarella A. (2018). Estrogen, angiogenesis, immunity and cell metabolism: Solving the puzzle. Int. J. Mol. Sci..

[33] Kang Y.J., Oh J.H., Seok H., Jo Y.Y., Kim D.W., Garagiola U., Choi J.Y., Kim S.G. (2020). 4-Hexylresorcinol exhibits different characteristics to estrogen. Appl. Sci..

