# Supplementary material for: Increased Level of Vascular Endothelial Growth Factors by 4-hexylresorcinol is Mediated by Transforming Growth Factor-β1 and Accelerates Capillary Regeneration in the Burns in Diabetic Animals
Source: Int J Mol Sci. 2020 May 14;21(10):3473. doi: 10.3390/ijms21103473 (PMC7279008; doi:10.3390/ijms21103473)
Supplement: Supplementary file 1 [file ijms-21-03473-s001.pdf]

International Journal of *Molecular Sciences*

A

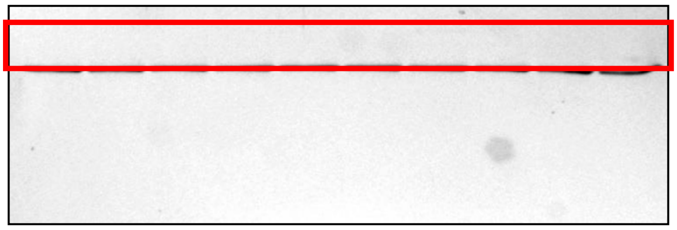

B

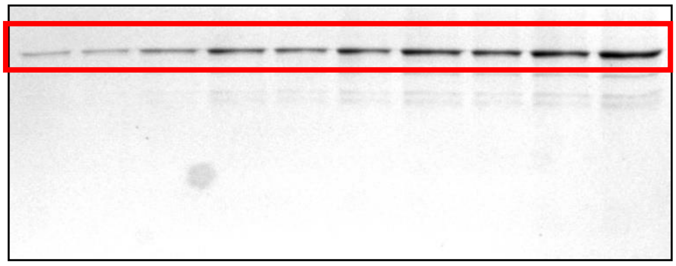

C

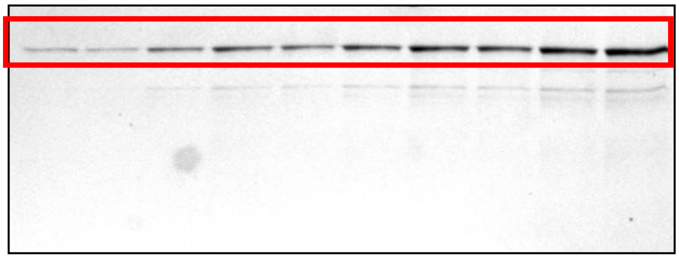

D

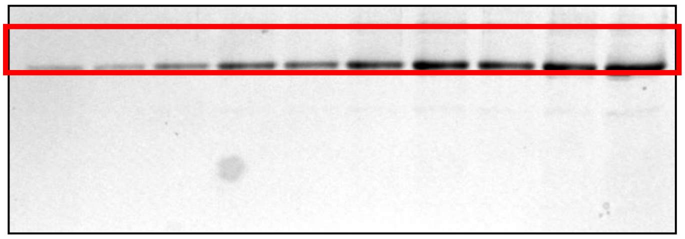

Figure S1. Full length blot of Figure 1A. The application of 4HR increased the expression of VEGF-A, VEGF-C, and TGF-β1. (**A**) β-actin (from left lane to right, **1:** no-treatment, **2:** 1 μg/mL 4HR at 2 h, **3:** 5 μg/mL 4HR at 2 h, **4:** 10 μg/mL 4HR at 2 h, **5:** 1 μg/mL 4HR at 8 h, **6:** 5 μg/mL 4HR at 8 h, **7:** 10 μg/mL 4HR at 8 h, **8:** 1 μg/mL 4HR at 24 h, **9:** 5 μg/mL 4HR at 24 h, **10:** 10 μg/mL 4HR at 24 h). (**B**) VEGF-A (from left lane to right, **1:** no-treatment, **2:** 1 μg/mL 4HR at 2 h, **3:** 5 μg/mL 4HR at 2 h, **4:** 10 μg/mL 4HR at 2 h, **5:** 1 μg/mL 4HR at 8 h, **6:** 5 μg/mL 4HR at 8 h, **7:** 10 μg/mL 4HR at 8 h, **8:** 1 μg/mL 4HR at 24 h, **9:** 5 μg/mL 4HR at 24 h, **10:** 10 μg/mL 4HR at 24 h). (**C**) VEGF-C (from left lane to right, **1:** no-treatment, **2:** 1 μg/mL 4HR at 2 h, **3:** 5 μg/mL 4HR at 2 h, **4:** 10 μg/mL 4HR at 2 h, **5:** 1 μg/mL 4HR at 8 h, **6:** 5 μg/mL 4HR at 8 h, **7:** 10 μg/mL 4HR at 8 h, **8:** 1 μg/mL 4HR at 24 h, **9:** 5 μg/mL 4HR at 24 h, **10:** 10 μg/mL 4HR at 24 h). (**D**) TGF-β1 (from left lane to right, **1:** no-treatment, **2:** 1 μg/mL 4HR at 2 h, **3:** 5 μg/mL 4HR at 2 h, **4:** 10 μg/mL 4HR at 2 h, **5:** 1 μg/mL 4HR at 8 h, **6:** 5 μg/mL 4HR at 8 h, **7:** 10 μg/mL 4HR at 8 h, **8:** 1 μg/mL 4HR at 24 h, **9:** 5 μg/mL 4HR at 24 h, **10:** 10 μg/mL 4HR at 24 h).

A

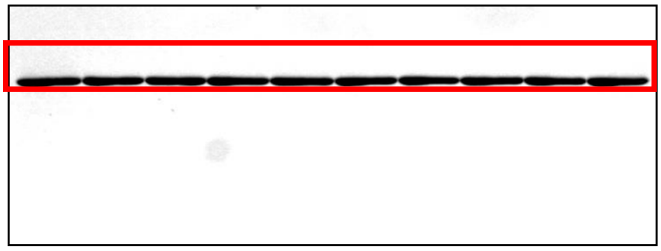

B

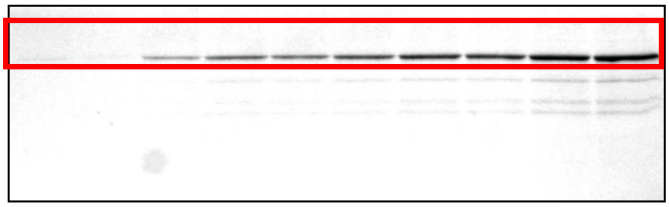

C

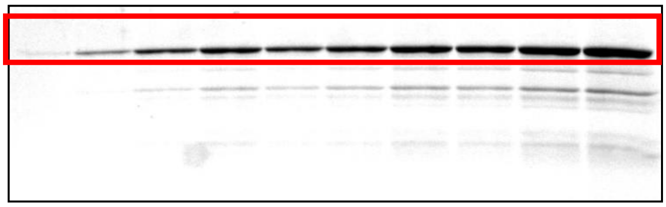

Figure S2. Full length blot of Figure 1B. The application of TGF-β1 increased the expression of VEGF-A and VEGF-C. (**A**) β-actin (from left lane to right, **1:** no-treatment, **2:** 1 ng/mL TGF-β1 at 2 h, **3:** 5 ng/mL TGF-β1 at 2 h, **4:** 10 ng/mL TGF-β1 at 2 h, **5:** 1 ng/mL TGF-β1 at 8 h, **6:** 5 ng/mL TGF-β1 at 8 h, **7:** 10 ng/mL TGF-β1 at 8 h, **8:** 1 ng/mL TGF-β1 at 24 h, **9:** 5 ng/mL TGF-β1 at 24 h, **10:** 10 ng/mL TGF-β1 at 24 h). (**B**) VEGF-A (from left lane to right, **1:** no-treatment, **2:** 1 ng/mL TGF-β1 at 2 h, **3:** 5 ng/mL TGF-β1 at 2 h, **4:** 10 ng/mL TGF-β1 at 2 h, **5:** 1 ng/mL TGF-β1 at 8 h, **6:** 5 ng/mL TGF-β1 at 8 h, **7:** 10 ng/mL TGF-β1 at 8 h, **8:** 1 ng/mL TGF-β1 at 24 h, **9:** 5 ng/mL TGF-β1 at 24 h, **10:** 10 ng/mL TGF-β1 at 24 h). (**C**) VEGF-C (from left lane to right, **1:** no-treatment, **2:** 1 ng/mL TGF-β1 at 2 h, **3:** 5 ng/mL TGF-β1 at 2 h, **4:** 10 ng/mL TGF-β1 at 2 h, **5:** 1 ng/mL TGF-β1 at 8 h, **6:** 5 ng/mL TGF-β1 at 8 h, **7:** 10 ng/mL TGF-β1 at 8 h, **8:** 1 ng/mL TGF-β1 at 24 h, **9:** 5 ng/mL TGF-β1 at 24 h, **10:** 10 ng/mL TGF-β1 at 24 h).

A

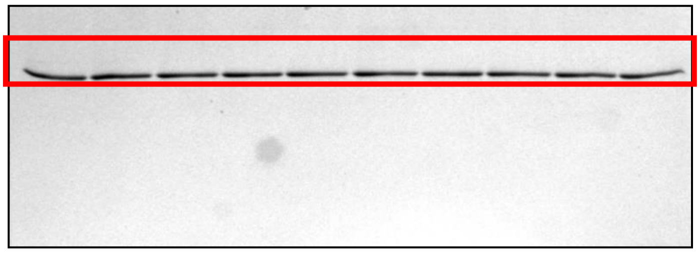

B

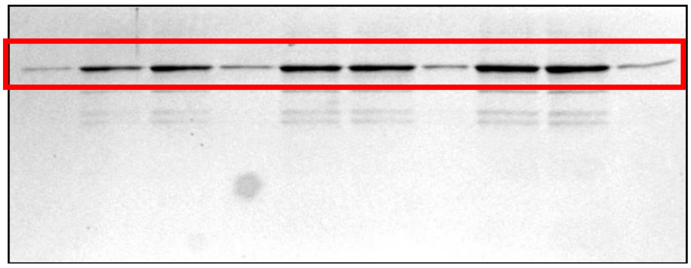

C

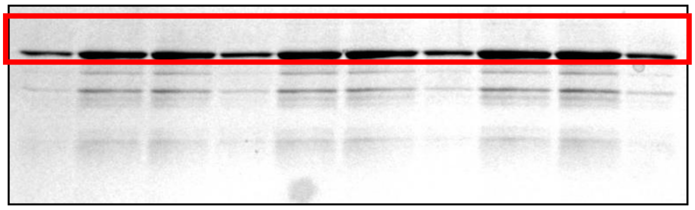

Figure S3. Full length blot of Figure 2A. The application of TGF-β1 siRNA inhibited the expression of VEGF-A, VEGF-C, which was induced by 4HR application. (A) β-actin (from left lane to right, 1: no-treatment, 2: 1 μg/mL 4HR, 3: 1 μg/mL 4HR with random RNA, 4: 1 μg/mL 4HR with TGF-β1 siRNA, 5: 5 μg/mL 4HR, 6: 5 μg/mL 4HR with random RNA, 7: 5 μg/mL 4HR with TGF-β1 siRNA, 8: 10 μg/mL 4HR, 9: 10 μg/mL 4HR with random RNA, 10: 10 μg/mL 4HR with TGF-β1 siRNA). (B) VEGF-A (from left lane to right, 1: no-treatment, 2: 1 μg/mL 4HR, 3: 1 μg/mL 4HR with random RNA, 4: 1 μg/mL 4HR with TGF-β1 siRNA, 5: 5 μg/mL 4HR, 6: 5 μg/mL 4HR with random RNA, 7: 5 μg/mL 4HR with TGF-β1 siRNA, 8: 10 μg/mL 4HR, 9: 10 μg/mL 4HR with random RNA, 10: 10 μg/mL 4HR with TGF-β1 siRNA). (C)VEGF-C (from left lane to right, 1: no-treatment, 2: 1 μg/mL 4HR, 3: 1 μg/mL 4HR with random RNA, 4: 1 μg/mL 4HR with TGF-β1 siRNA, 5: 5 μg/mL 4HR, 6: 5 μg/mL 4HR with random RNA, 7: 5 μg/mL 4HR with TGF-β1 siRNA, 8: 10 μg/mL 4HR, 9: 10 μg/mL 4HR with random RNA, 10: 10 μg/mL 4HR with TGF-β1 siRNA).

A

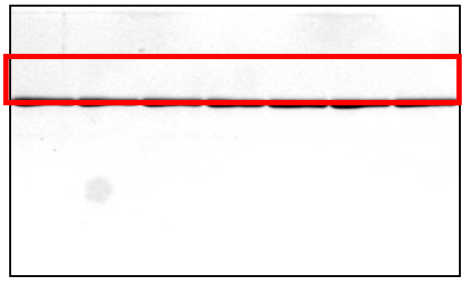

B

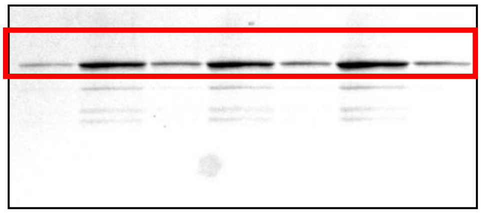

C

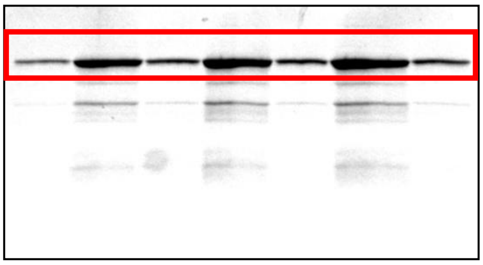

Figure S4. Full length blot of Figure 2B. ALK5 inhibitor (A83-01) showed a similar effect to that of TGF-β1 siRNA on VEGFs expression by 4HR in HUVECs. (A) β-actin (from left lane to right, **1:** no-treatment, **2:** 1 μg/mL 4HR, **3:** 1 μg/mL 4HR with 12 nM A83-01, **4:** 5 μg/mL 4HR, **5:** 5 μg/mL 4HR with 12 nM A83-01, **6:** 10 μg/mL 4HR, **7:** 10 μg/mL 4HR with 12 nM A83-01). (B) VEGF-A (from left lane to right, **1:** no-treatment, **2:** 1 μg/mL 4HR, **3:** 1 μg/mL 4HR with 12 nM A83-01, **4:** 5 μg/mL 4HR, **5:** 5 μg/mL 4HR with 12 nM A83-01, **6:** 10 μg/mL 4HR, **7:** 10 μg/mL 4HR with 12 nM A83-01). (**C**) VEGF-C (from left lane to right, **1:** no-treatment, **2:** 1 μg/mL 4HR, **3:** 1 μg/mL 4HR with 12 nM A83-01, **4:** 5 μg/mL 4HR, **5:** 5 μg/mL 4HR with 12 nM A83-01, **6:** 10 μg/mL 4HR, **7:** 10 μg/mL 4HR with 12 nM A83-01).

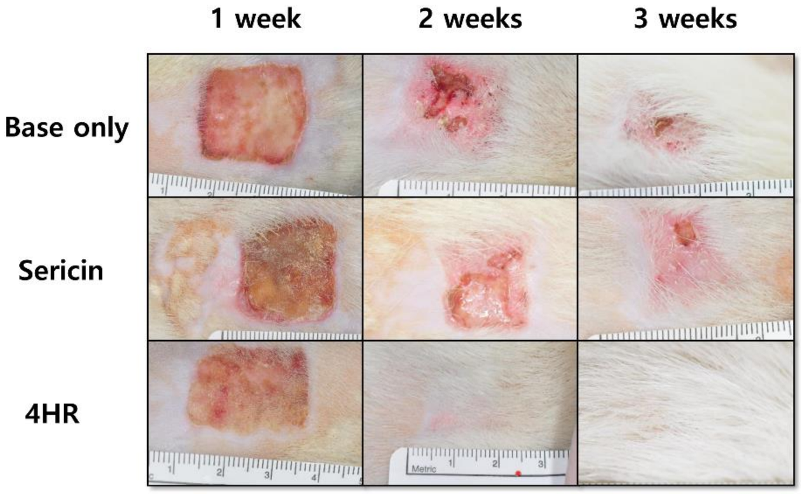

Figure 5. The images for burn wound at different time point.

A

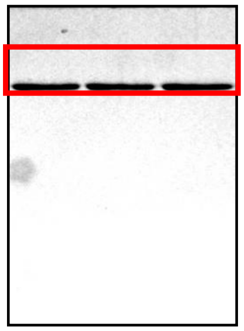

B

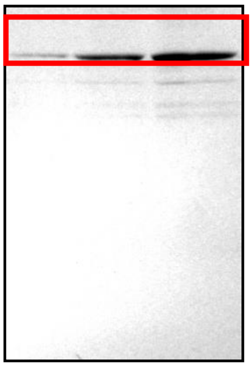

C

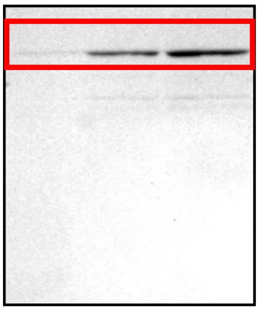

D

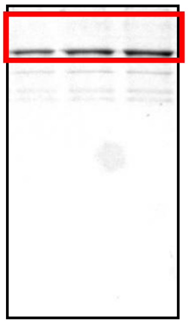

E

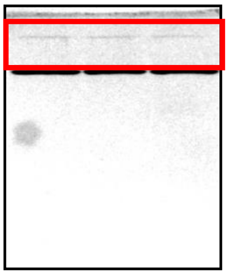

F

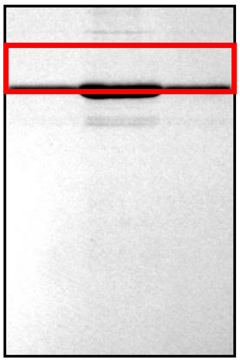

G

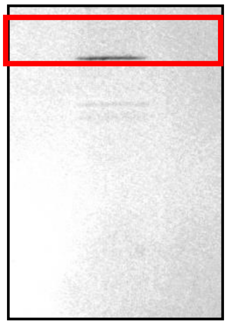

H

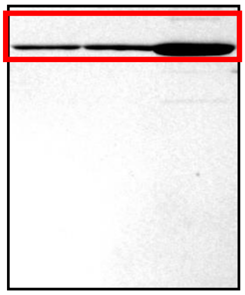

Figure S6. Full length blot of Figure 5. (**A**) β-actin (from left lane to right, **1:** ointment base only, **2:** sericin ointment, **3:** 4HR ointment). (**B**) TGF-β1 (from left lane to right, **1:** ointment base only, **2:** sericin ointment, **3:** 4HR ointment). (**C**) VEGF-A (from left lane to right, **1:** ointment base only, **2:** sericin ointment, **3:** 4HR ointment). (**D**) VEGF-C (from left lane to right, **1:** ointment base only, **2:** sericin ointment, **3:** 4HR ointment).

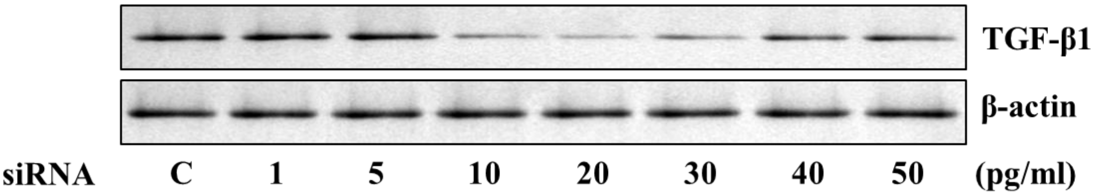

Figure S7. Determination of optimal concentration of TGF-β1 siRNA. The optimal concentration of TGF-β1 siRNA was determined as 20 pg/mL.

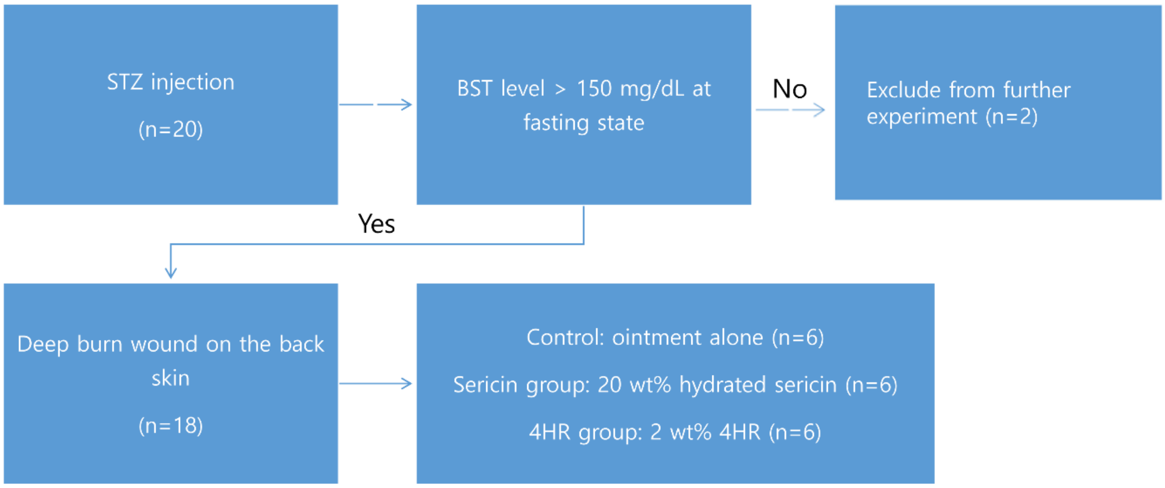

Figure S8. CORT chart for the animal experiment.
